# Acute hemorrhagic leukoencephalitis Associated with COVID-19

**DOI:** 10.1590/0004-282X-ANP-2021-0319

**Published:** 2021-12-17

**Authors:** Daniel Teixeira Dos Santos, Wyllians Vendramini Borelli, Clarissa Both Pinto, Iuri Christmann Wawrzeniak, Marino Muxfeldt Bianchin, Juliana Avila Duarte

**Affiliations:** 1 Hospital de Clínicas de Porto Alegre, Porto Alegre RS, Brazil.; 2 Universidade Federal do Rio Grande do Sul, Programa de Pós-Graduação em Ciências Médicas, Porto Alegre RS, Brazil.; 3 Universidade Federal do Rio Grande do Sul, Medicina Interna, Disciplina de Radiologia, Porto Alegre RS, Brazil.

**Figure 1:**
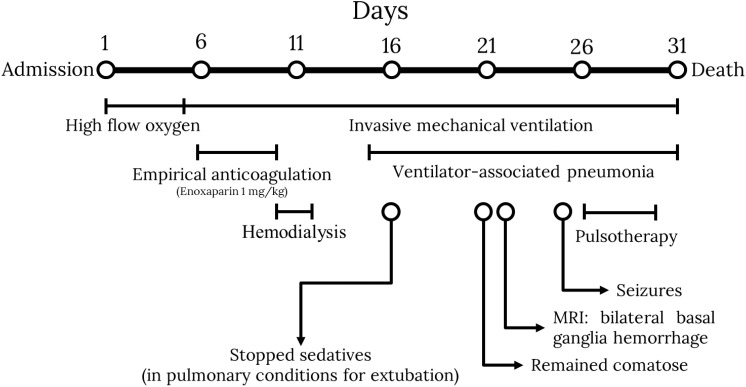
Hospital events timeline.

**Figure 2:**
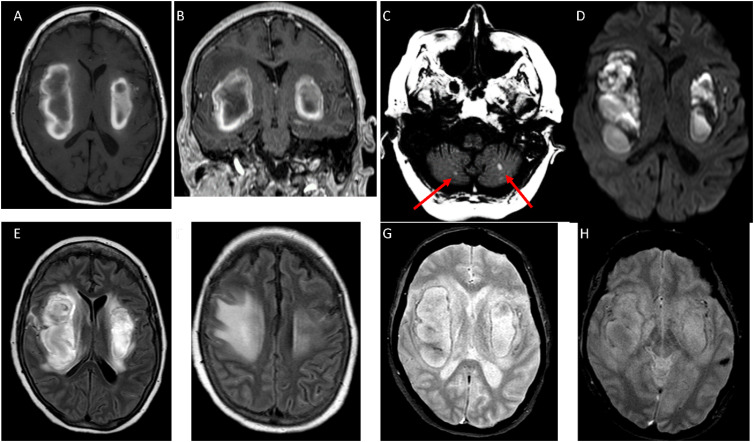
(A) Axial T1 spin eco (SE) showed central hypointense with peripheral hyperintense lesions on bilateral deep white matter with effacement of the lateral and third ventricles. (B) Coronal reconstruction of a volumetric T1 gradient-echo (GRE) showed central hypointense with peripheral hyperintense lesions on deep white matter. (C) Axial T1 SE showed two small hemorrhages on both cerebellar hemispheres (red arrows). (D) Diffusion-weighted imaging revealed possible restriction on bilateral deep white matter compatible with subacute hemorrhages. (E): Axial FLAIR at basal ganglia level showed hyperintensity lesions on bilateral deep white matter with surrounding edema. (F) Axial FLAIR at supraganglionic level showed extensive bilateral edema. (G): Axial T2* at basal ganglia level showed hyperintensity lesions on deep white matter with hypointensity due to subacute hemorrhages. (H) Axial T2* at lower basal ganglia level showed hyperintense lesions on bilateral deep white matter with hypointensity with blooming effect due to subacute hemorrhages and there was another small hemorrhage on the left temporal lobe.

A 64-year-old woman with diabetes and heart failure presented with severe coronavirus disease 2019 (COVID-19), remaining comatose after sedation withdrawal (Figure 1). Magnetic resonance imaging (MRI) showed bilateral subacute hematomas in the white matter, with significant mass effect (Figure 2). She was treated with high-dose intravenous methylprednisolone, but developed seizures, pulmonary bacterial infection, and ultimately died.

Acute hemorrhagic leukoencephalopathy has been previously reported in patients with COVID-19^1^, and it is a possible diagnosis for this patient. As our patient presented larger hematomas than previously described^2^ and received empirical anticoagulation due to a suspected pulmonary embolism, we speculate that an inflammatory process associated with SARS-CoV-2 could have been complicated by this therapy.
